# Poetry

**Published:** 1884-05

**Authors:** 


### PERPETUITY OF NATURE.

BY BULWER-LYTTON.

There is no death! The stars go down
To rise upon some fairer shore;
And bright in heaven's jewelled crown
They shine for ever more.

There is no death. The dust we tread
Shall change beneath the summer showers
To golden grain, or mellow fruit,
Or rainbow-tinted flowers.

The granite rocks disorganize,
To feed the hungry moss they bear;
The forest leaves drink daily life
From out the viewless air.

There is no death! The leaves may fall;
The flowers may fade and pass away;
They only wait through wintry hours,
The coming of the May.

There is no death! An angel form
Walks o'er the earth with silent tread;
He bears our best-loved things away,
And then we call them "dead."

### *SPRING.*

*Why twit me with my passing years?*
*The tide that flows is bound to ebb,*
*And life is but a stream of tears,*
*And pleasure but a spider's web.*

*The friends that come, the frieuds that go,*
*Are but the motes the sunbeams bless,*
*And enemies are drifts of snow*
*That melt before forgetfulness.*

*And so with Springs. They take their birth*
*When all the ice of Winter's burst;*
*A moment's gladness fills the earth,*
*But Summer's heat turns them to dust.*

